# Hypodermic Medication

**Published:** 1874-02

**Authors:** 


Editor Atlanta Medical and Surgical Journal: In the January, 1874, number of your valuable journal, page 604, I see a few thoughts suggested relative to the endermic administration of the acetate of morphia.

You ask for the experince of readers in the future use of that method. I have no experience to offer, and probably will not have, unless the physiological phenomena of irritation, inflammation, and absorption undergo a radical revolution in the minds of scientists.

I regard the proposed medication as horrid and barbarous torture, inasmuch as the only object to be attained is simply the introduction of a little acetate of morphia under the skin.

The amount of moisture to which a certain length of the thread is subjected must necessarily be very slight, and when it is remembered that it takes several minutes for the hydrated section to take up the medicine, it appears reasonable that the time of its supposed absorption would occupy a much longer period, inasmuch as acetate of morphia is less soluble in blood serum than in water.

It is a well known fact, that the moment a foreign body, such as we speak of, is placed in the tissue, just so soon is an effort made by nature to expel it, capillaries are more or less torn and lacerated, a solution of continuity among the adipose vesicles, a displacement of nerve filaments, and a pressure made on the contiguous parts to make room for the thread; in other words it is a seton, the pathological effects of which are understood. Admit, for instance, that it will effect the object for which it is intended, it will necessarily take several minutes for the medicine to be absorbed to say nothing of the pain it would produce; the thread will certainly produce more or less irritation by reason of its presence, the blood stasis it would cause, the laceration of nerves, vesicles and blood vessels, and the residue of capillary fibres from the string, all of which would beget congestion, induration, and inflammation.

I believe that the morphine would not be absorbed to any satisfactory extent, but that the physiological processes of elimination would promptly begin.

Another reason why it should not be used, is, that it would be shocking to the sensibilities of almost any patient, the idea of *sewing* medicine into their flesh. The hypodermic syringe is fearful enough for them, but if we advance, armed with a needle and thread, we would gain the victory by a precipitate and hasty retreat.

Respectfully,

EUFAULA, ALA., Janury, 1874. Z. T. DANIEL, M.D.

We insert, with pleasure, the foregoing letter of our correspondent, and still think the proposal worthy of further expression of opinion as well as observation from other members of the profession. We feel quite sure that there will be both physicians and patients, who will differ radically with our correspondent in the position that the proposed substitution of a medicated thread and cambric needle for the hypodermic syringe is "horrid and barbarous torture."

We this month present the original article, in *Our Exchanges*, which pointed our suggestions, and which was crowded out of our last issue. The *experimentum crucis* can alone decide the value of the proposed method of hypodermic medication.

